# The *Accessibility* and the *Digital Divide* in the Apps during the COVID-19. Comment on Cao et al. The Impact of Using mHealth Apps on Improving Public Health Satisfaction during the COVID-19 Pandemic: A Digital Content Value Chain Perspective. *Healthcare* 2022, *10*, 479

**DOI:** 10.3390/healthcare10071252

**Published:** 2022-07-05

**Authors:** Daniele Giansanti, Antonia Pirrera, Paola Meli

**Affiliations:** Centre Tisp, The Italian National Institute of Health, 00161 Rome, Italy; antonia.pirrera@iss.it (A.P.); paola.meli@iss.it (P.M.)

We are writing to you as the corresponding author of the interesting study “The Impact of Using mHealth Apps on Improving Public Health Satisfaction during the COVID-19 Pandemic: A Digital Content Value Chain Perspective” [1].

With this correspondence, through two *points of view*, we intend to, *first* recall, through a short analysis, the merits and added value of your study set in the Special Issue “The Impact of Mobile Technology in the Battle against COVID-19: Successes and Failures” https://www.mdpi.com/journal/healthcare/special_issues/COVID_Mobile (accessed on 5 May 2022) [2,3]. *Second*, reflect with you on some important aspects, such as the accessibility and the *digital divide*, which have an impact on the topic you are addressing and which we believe can be of scientific interest for future insights and research.

We found that this is a work particularly stimulating and that gives a great added value in the field and in particular in the Special Issue.

Specifically, we believe that this study has the great merit of, at the same time: (a) focusing on important key aspects of the support of the mHealth during the COVID-19 pandemic; (b) highlighting the opportunities and the potentialities; (c) proposing a useful model to assess the impact of this technology in mHealth; and (d) giving a quantitative assessment, which is very useful both for the current period and in perspective.

Specifically, we agree with the authors that this study has both theoretical and practical implications.

*From a theoretical point of view* this study: (I) not only verified the mHealth Apps’ functional value from an empirical perspective but also demonstrated the emotional and societal values for public health governance during the COVID- pandemic and the positive impact between user-doctor. satisfaction. (II) Further refines the functional, emotional, and social values in the Digital Content (DC) value chain framework in specific contexts, thus expanding the dimensions of value creation in DC and enriching the antecedents that influence the interactions between users and DC.

*From a practical point of view* this study has three useful suggestions: (I) It enriches mHealth Apps and expands the scope of application to guarantee the basic medical needs of users. In COVID-19, recommendation algorithms can be cleverly used to give the most appropriate results and analyze suggestions based on the questions submitted by users and suggest and associate with related functions. (II) It is necessary to provide users with healthcare confidence during the COVID-19 pandemic. This requires the governance bodies to formulate effective epidemic prevention measures in line with public opinion and pass it onto society through the efficient information distribution function of mHealth apps. Also, the self-assessment Apps could be useful to monitoring these perceptions, trends, and opinions. In this case the gamification could be useful. (III) It is also necessary to establish a harmonious doctor–patient relationship in the. These mHealth Apps must be designed to allowing and facilitating this.

The whole study is both interesting and comprehensive. There is no criticism. There are many insights into the future based on what emerged in the study, during the pandemic, specifically in the Special Issue [2,3] and on how mHealth and other apps should consider.

We have noted that two problems in the Apps use, that can be also interconnected with each other, which were already present before the pandemic were often referred to in this period.

A first problem is the ‘digital divide’. The digital divide regards the gap between those who have effective access to information technologies and those who are partially or totally excluded from it. The digital divide has three sides. The first is the difficulty in the access to the infrastructures [4]. The second level is represented by the literacy of people with the technologies [5]. The third level is represented by the potential benefit level in terms of economic, cultural, social, and personal types of using the technologies [6]. During the COVID-19 the Digital Divide was evident in all of the three levels and has been exacerbated [7,8,9,10].

A second problem is the accessibility of disabled people to these technologies [11]. Disabilities are of various nature, forms, and complexity (auditory, cognitive, neurological, physical, speech, visual). The new software development policies are increasingly, rightly, pushing towards a design of citizen-oriented systems that are more accessible to disabled people [12]. Designers and institutions must increasingly take this into account.

This also applies to mHealth and Apps. Digital Divide and accessibility are linked. For example, also benefits of accessibility [12]:

- People using mobile phones, smart watches, smart TVs, and other devices with small screens.
- Older people with changing abilities due to ageing with the risk of moving away from technologies,
- People with “temporary disabilities”,
- People with “situational limitations”,
- People using a slow Internet connection due to limited resources.

Accessibility and the Digital Divide are two important and interrelated aspects that governments and designers must increasingly consider when it comes to the use of technologies and the accessibility of citizens for the purposes of monitoring and disseminating information.

This is particularly true when we talk about healthcare where Apps and mHealth can give an important added value as you have fully illustrated in your study [1].

Considering these factors and your study we would like to discuss this with you and have a reflection as a reply. We believe that this would be of great added value for the Special Issue and would further enrich it.
